# Development of the Adolescent Preoccupation with Screens Scale

**DOI:** 10.1186/s12889-017-4657-1

**Published:** 2017-08-11

**Authors:** Simon C. Hunter, Stephen Houghton, Corinne Zadow, Michael Rosenberg, Lisa Wood, Trevor Shilton, David Lawrence

**Affiliations:** 1 School of Psychological Sciences and Health, University of Strathclyde, Glasgow, Scotland; 2 Graduate School of Education, The University of Western Australia, Perth, Australia; 3 Health Promotion Evaluation Unit, The University of Western Australia, Perth, Australia; 4 School of Population Health, The University of Western Australia, Perth, Australia; 5 National Heart Foundation, The University of Western Australia, Perth, Australia

**Keywords:** Screen use, Pre-occupation, Problematic, Adolescents

## Abstract

**Background:**

Although public health concerns have been raised regarding the detrimental health effects of increasing rates of electronic screen use among adolescents, such effects have been small. Instruments currently available tend to be lengthy, have a clinical research focus, and assess young people’s screen use on specific screen-based activities (e.g., TV, computer, or internet). None appear to address screen use across a broad range of screens, including mobile devices and screen-based activities. The objective was to develop a new and short self-report scale for investigating adolescents’ screen use across all screens and screen-based activities in non-clinical settings.

**Methods:**

The Adolescent Preoccupation with Screens Scale (APSS) was developed over a three stage process. First, a review of the current literature and existing instruments was undertaken and suitable items identified. Second, the draft APSS was piloted with adolescents and item affectivity and discrimination indices were calculated. Third, a cross sectional school based online survey of 1967 Australian adolescents in grades 5 (10 years old), 7 (13 years) and 9 (15 years) from 25 randomly selected schools was conducted.

**Results:**

Factor Analysis on a sub-sample of the data (*n* = 782) and Confirmatory Factor Analysis on the remaining sub-sample (*n* = 1185), supported a two-factor model. The first factor reflects adolescents’ mood management with screen use, and the second reflects a behavioural preoccupation. The measure demonstrated strong invariance across sex and across Grades 5, 7, and 9. Both factors displayed good internal consistency (α = .91 and .87, respectively). Sex and grade differences on both scales were investigated and boys in Grade 5 reported higher levels of both mood management and behavioural preoccupation with screens. There were no sex differences on mood management in Grades 7 and 9, but girls reported higher behavioural preoccupation in both these later grades.

**Conclusion:**

The APSS provides researchers with a new, brief and robust measure of potentially problematic screen use across a wide array of screens, including mobile devices, so readily accessed during adolescence.

## Background

Mobile devices now make screen use an important part of young people’s social and academic lives and provide unprecedented access to the wider world across a variety of activities [1]. Research demonstrates that screen media time (SMT) increases with age (see [2, 3]), especially during adolescence [3, 4]. Long-held guidelines recommending two-hour limits on screen time [5] have now been superseded by the recommendation that parents develop personalised media plans with their children to place consistent limits on screen time [6]. However, consistency may be 8 h of daily screen time for some, while for others it might be considerably less.

The extent to which SMT is associated with problematic behavior and/or maladjustment is the subject of much debate in the scholarly literature. For example, higher levels of SMT have been associated with problematic sleep patterns, including quality and efficiency of sleep, and sleep onset latency [7]. Furthermore, regardless of the developmental stage of the individual (e.g., 3 year olds to 17 year olds) these SMT related sleep disturbances have been linked to higher levels of behavioral health problems (i.e., internalizing, externalizing, and peer problems) [8]. Poorer dietary choices [9] and in turn obesity [10] have also been linked with increased SMT. A significant positive association has also been found between duration of screen time and severity of depression and anxiety, while video game playing and computer use (but not TV viewing) has been linked with severity of depressive symptoms. Video game playing has also been associated with severity of anxiety [11]. Research [12] has reported that low to moderate screen use was no worse than screen abstinence across a range of negative outcomes and that TV/computer game use had to exceed 6 h per day before significant effects were observed. Even at this level of screen use though, only very small effects were reported.

The extent to which screen time is associated with these physical and mental health outcomes in children and adolescents is unclear however, and significant effects have been reported by some to be small. For example, one recent meta-analysis suggested the effects of video game use on aggression, prosocial behaviour, depressive symptomatology, academic performance, and attention deficit problems are only very small [13], while a 1-year longitudinal study of video game players aged 14 to 21 years found that the use of violent video games was not a substantial predictor of physical aggression, at least in the later phases of adolescence [14]. A second meta-analysis concluded that TV and video game use is not associated with body fatness [15]. In addition to small effects, a number of methodological flaws common to significant portions of this literature have also been highlighted (e.g., failure to pretest, use of unstandardized measures, possible effects of researcher degrees of freedom, etc) [13].

While there is evidence showing both positive and negative behavioural and health related outcomes associated with SMT, a separate meta-analysis suggested the relationship was not straightforward [16]. Specifically, beneficial effects of screen time were evidenced at 1 h per day, but negative effects when over 2 h per day. In a separate report of four linked studies [17], critical cut-offs also had to be reached before negative effects were observed, though the authors cautioned that any effects were again small.

When seeking to understand how, when, and if screen use is problematic/harmful, it may be more important to consider screen use **not** as “time spent using a screen” but rather the degree to which it becomes something a young person is overly-dependent upon. While this links to the notion of addiction, it is important to be aware that the body of evidence conceptualising screen use in such a fashion is controversial (e.g., [18, 19]). Evidence regarding potentially excessive or problematic dependence upon screens has been obtained from studies primarily focusing on one or two specific forms of screen use and using instruments specific to those forms [20]. For example: TV viewing [21], Internet use [22–25], mobile phone use [26, 27], Facebook use [28], or computer use [29]. Measures have also tended to assess a single factor (e.g., [21, 22]), though two [20], three [23], and even six [24] have been reported. Although the number of items across the measures have ranged from two [21] to 27 [26], most are long and are developed for clinical research [30]. What these studies indicate is that important themes in young people’s screen use relate to coping/emotion management, mood modification, preoccupation with screens, escaping problems, and impulse control.

We propose that assessment of such a construct for screen use is important because it may act as a moderator of the effects of screen use. Specifically, screen use may be most problematic when it is an activity that an individual is overly reliant upon, especially if that over-reliance involves the use of screens to cope with problems they are facing. The current study set out to present data on the development of a measure to assess behaviors reflecting preoccupation with screen activity. The objective was to draw on existing measures relating to more discrete forms of screen use to develop a new online self-report scale which can be used by researchers to investigate young people’s preoccupied screen use across **all** screens and screen-based activities. This is an important issue considering the extent to which young people multi-task on screens [1, 31].

## Methods

### Phase I: item generation

Following an examination of the literature, a review was undertaken to identify instruments currently available that reflect problematic and potentially excessive screen use. In total, 10 instruments were identified for further investigation: The Dispositional Media Use Motive Scale [32], Assessment of Computer Game Addiction in Children – Revised [33], Pathological Video-gaming Scale [34], Problem Video Game Playing Scale [35], Pathological Gaming Scale [29], Problematic Online Game Use Scale [36], Game Addiction Scale for Adolescents [37], Young’s Internet Addiction Scale [38], Compulsive Internet Use Scale [39], and the Mobile Phone Problem Use Scale [40]. To be included, instruments had to have been used in research published in peer reviewed journals, be appropriate for use with adolescents, and be in a form that could be modified to reflect screen use per se.

A panel of three, with expertise in adolescent psychology and measurement, then reviewed the instruments to identify items for possible inclusion in the new (APSS) short measure. In total, 48 items were identified for possible inclusion in the new scale. These items were subsequently reduced to 36 as a result of the panel identifying and removing duplicate items and items not appropriate for use with adolescents. Each item was modified to be in an online self-report age-appropriate format and to reflect screen media use per se, rather than an individual’s engagement with a specific screen (e.g., TV, internet, or videogame playing). Responses were on a 1–6 scale (1 = Never, 2 = Rarely, 3 = Sometimes, 4 = Often, 5 = Very often, 6 = Always).

### Phase II: preliminary validation of the APSS

Permission to conduct the research was initially obtained from the Human Research Ethics Committees of the University of Western Australia and the Western Australian State Department of Education (Evaluation and Accountability Directorate). The initial draft of the APSS was administered to 10 young people (ages 10 to 16 years) located in low to high socioeconomic status areas as indexed by their Socio-Economic Index for Areas (SEIFA), Australia, 2011 [41]. These individuals, recruited via teacher contacts, were asked to comment on the face and content appropriateness of the 36 items and the response option format. This led to the modification of one item (inserting “worried” to clarify the meaning of “anxious”). Other feedback was very positive with all participants reporting that the online APSS was easy to access, understand and complete.

Following this, 28 young people (14 male and 14 female) randomly selected from one primary (*n* = 16) and one high (*n* = 12) school in low to middle socioeconomic status areas as indexed by their SEIFA [41] completed the online APSS. The item affectivity in the sample of the 36 items varied from .23 to .62, meeting the recommended item affectivity range (>.2 *-* < .8) [42], and all were therefore retained. Five items had a discriminatory power of less than .3, with three showing negative correlations. Affectivity and discrimination indices were then examined concurrently, along with theoretical importance to the construct. Five items were subsequently removed, reducing the item pool to 31.

### Phase III: factorial structure of the APSS

#### Participants and settings

In total, 1967 adolescents (1034 male, 918 female, 15 did not report) from Grades 5 (10–12 years old, *n* = 612), 7 (12–14 years old, *n* = 690), and 9 (14–16 years old, *n* = 665) took part. Initially, 30 schools in Western Australia were randomly selected from metropolitan and rural areas and of these 25 agreed to participate. Of the 25, 14 were state government primary schools (four in rural locations), six were state government high schools (three in rural locations), one was a state government District high school (rural location catering for grades K to 10) and four were non-government private schools (K – 12). Participating schools represented varied socio-economic status (SES) areas as indexed by their SEIFA [41]. Seven primary schools were in low SES areas, four in mid SES areas, and three were in high SES areas. Of the six state government high schools, three were in low SES areas and three were in mid SES areas. The District High school was in a low SES area and of the four private non-government high schools three were in high SES areas and the remaining one was in a mid SES area. To recruit the sample, information sheets and consent forms were sent to the parents of students in the specified school grade levels requesting permission for their son/daughter to participate. Students were also asked for written consent. Students providing two consenting signatures were included in the sample.

## Instrumentation

All data were collected using the online survey software Qualtrics. On accessing the APSS participants were asked to provide basic biographical information (school grade level, sex, date of birth). Then the following text was prominently displayed “*Screens can mean anything that shows a picture that you watch or interact with. Below are some pictures of screens you may use. These include an iPod Touch, iPad, Mobile Phone or iPhone, TV, Laptop Computer, Portable PlayStation or an Xbox.* (Images of these screens were then presented.) *Examples of things you can do on screens are watch TV, search the internet, use social networking sites, use instant messenger, send and receive emails, play games, online shopping, download music, do school work and homework, and watch music videos”*. Participants were told to think about their screen use from the time of waking up (including before school, during school, after school, at home or at a friend’s house, and in the evening) until going to bed. They were then instructed to “*Keep thinking about what screens you use and how you feel when you use them. How strongly do you agree or disagree with the following statements about* ***your*** *screen use*? The 31 items comprising the APSS were then presented, and participants responded either Never (scored 1), Rarely (2), Sometimes (3), Often (4), Very often (5), or Always (6) to each item.

## Procedure

Permission to conduct the research was obtained from the Human Research Ethics Committees of the University of Western Australia and the Western Australian State Department of Education (Evaluation and Accountability Directorate). The 25 schools who expressed an interest in participating received information sheets explaining the research, along with a follow-up phone call to answer any questions and to finalise their involvement. Information sheets and consent forms (for active informed consent from parents) were sent to the parents of students in Grades 5, 7 and 9. The sample of 1967 students represented a participation rate of approximately 71%.

The APSS was administered to the participants in groups of 15–25 during a 4 week period when the electronic link remained open. A unique code was given to all participants so that they could access the online APSS to complete in confidence. School staff led data collection sessions and written instructions were provided to ensure standardization of procedures and to address any technical difficulties should they arise. On average the APSS took approximately 15–20 min to complete.

## Statistical analyses

Our first analysis included a maximum-likelihood Exploratory Factor Analysis (EFA) with promax rotation. This was followed by a Confirmatory Factor Analysis (CFA). Cronbach’s coefficient alpha was used to assess internal reliability in both sets of analyses. The factorial invariance of the CFA model across sex and across school grade was then assessed by comparing three models. To assess grade and sex differences in APSS scale scores, factor score weights were calculated. These scores were then used as dependent variables in a two-way independent ANOVA with sex and grade entered as independent variables. Bonferroni corrected *t*-tests were carried out to compare boys and girls at each grade level. Correlations between the sub-scales were calculated.

## Results

Exploratory Factor Analyses: An EFA was conducted using maximum-likelihood factor analysis with promax rotation with a random split sub-sample of the data (*n* = 782) using SPSS 23.0. This produced a six-factor solution where the first factor accounted for 41.04% of the variance, and the remaining five accounted for between 3.24% and 5.28% each. Inspecting these, a two- or three-factor solution seemed most conceptually plausible. Next, we forced a two-factor solution and a three-factor solution on the data. The two-factor solution produced factors on which most items loaded reasonably highly (>.4) and where there were relatively few cross-loading items. The three-factor solution produced similar factors, but the third factor reflected physical complaints associated with screen use, and only contained two items (“I get headaches from using screens”, “I get sore eyes from using screens”). Those two items were dropped and the two-factor solution was re-evaluated. The first factor accounted for 43.53% of the variance and the second factor 5.53%. From these items, we dropped five which did not clearly load on either of the two scales, i.e., those loading >.3 on both factors.

Examining the factor structure of the scales which resulted from this process revealed two well-defined factors, one concerning emotional issues connected with screen use (43.05% of the variance), which we named *Mood Management,* and one concerning time spent on screens (accounting for 6.57% of the variance), which we named *Behavioural Preoccupation*. We dropped three final items because conceptually they did not fit with the rest of the sub-scale with which they were associated. The final two-factor solution therefore comprised 21 items, with 13 items concerning *Mood Management* using screens (e.g., “I use screens to make myself feel better”) and 8 items concerning *Behavioural Preoccupation* with screens (e.g., “I spend too much time on screens”). The first sub-scale accounted for 43.93% of the variance and the second for 7.32%, and all items had loadings of .44 or above (see all items and factor loadings in Table 1). Internal reliability was good for both sub-scales (α_*Emotional*_ = .91; α_*Behavioural*_ = .87).

**Table 1:** Factor loadings from the exploratory factor analysis rotated factor matrix (and Factor Scores from Confirmatory Factor Analysis) for final 21 items

	Mood management	Behavioural preoccupation
I use screens to make myself feel better.	.91 (.085)	
I am less lonely when I am using screens.	.81 (.061)	
When I try to reduce my screen use I feel anxious (worried).	.74 (.097)	
When I am not using screens, I keep thinking about them.	.71 (.105)	
I feel closer to people who I know from using screen than people in the real world.	.69 (.035)	
I feel bad when I cannot use screens.	.68 (.099)	
When I am using screens, I feel like I am somewhere else.	.68 (.053)	
People I meet online using screens are easier to understand than real people.	.67 (.036)	
When I can’t use screens I get restless or irritable.	.67 (.143)	
I feel most pleasure when using screens.	.65 (.073)	
I use screens to forget about real life.	.60 (.061)	
I feel a buzz of excitement when on screens.	.52 (.043)	
People I meet online accept me better than those in real life.	.45 (.024)	
I spend too much time on screens.		.84 (.110)
I find myself thinking or saying “just a few more minutes” when using screens.		.76 (.075)
I stay on screens longer than I mean to.		.74 (.135)
I go to bed late because I have been using screens.		.74 (.071)
My parents complain that I use screens too much.		.73 (.054)
I have got into trouble with my parents because of using screens too much.		.65 (.050)
I lose track of time when I am using screens.		.59 (.091)
I use screens even if I have more important things to do.		.55 (.118)

Confirmatory Factor Analyses: A CFA on the 21-item two-factor EFA solution was conducted. The measurement model was assessed with AMOS23.0 using the remainder of the sample (*n* = 1185). Three fit indices assisted in providing evidence of the goodness-of-fit of the accepted model: the Comparative Fit Index (CFI), the Bentler-Bonett Normed Fit Index (NFI), and the Root Mean Square Error of Approximation (RMSEA) and its 90% confidence intervals. A value of .95 or greater for the CFI and the NFI is expected of models considered to be well fitting and above .90 reflects adequate fit. RMSEA values of less than .06 indicate a good fit [43, 44]. The model was not initially well-fitting: NFI = .866, CFI = .879, RMSEA = .080 (.077, .084). We evaluated items to consider whether there was a justification for correlating error terms, for example certain items may share a focus on a specific sub-set of issues. Based on this, we identified three items in the *Mood Management* sub-scale (“I feel closer to people who I know from using screens than people in the real world”, “People I meet online using screens are easier to understand than real people”, and “People I meet online accept me better than those in real life”) which all shared a clear, common theme and so we permitted their error terms to co-vary. We also identified two items on the *Behavioural Preoccupation* sub-scale (“My parents complain that I use screens too much”, and “I have got into trouble with my parents because of using screens too much”) and also allowed their error terms to co-vary. These model adjustments resulted in better, and adequate, fit: NFI = .922, CFI = .936, RMSEA = .059 (.056, .063).

The factorial invariance of the model across sex and across school grade was assessed by comparing three models. The first was an unconstrained model where the same factor structure was present for the competing groups (e.g., boys and girls) but no further constraints were placed upon the model. The second model was a weak factorial model, where the addition of constraints upon the factor loadings was added. Finally, the third model was a strong factorial model and included the additional constraint that indicator intercepts be equal. Strong factorial invariance indicates that slopes and intercepts are equal across groups, supporting the assertion that factor scores are comparable across groups [44]. We did not use change in chi-square as an indicator of invariance because of its documented sensitivity to sample size [44]. Rather, we used change in CFI (∆CFI) as one indicator of invariance (∆CFI > −.01 indicates violation of invariance) and whether the invariance model’s 90% RMSEA confidence intervals included the RMSEA point estimate of the unconstrained model [44]. These criteria indicated support for strong factorial invariance across both sex and grade (see Table 2).

**Table 2:** Fit indices used for assessing factor invariance across sex and grade

Model	CFI		∆CFI (vs. preceding model)		RMSEA (90% CI)	
	Sex	Grade	Sex	Grade	Sex	Grade
Model 1 (Unconstrained)	.927	.918	-	-	.045 (.042, .048)	.039 (.037, .042)
Model 2 (Weak invariance)	.927	.914	.000	−.004	.044 (.043, .047)	.039 (.037, .041)
Model 3 (Strong invariance)	.918	.900	−.009	−.014	.046 (.042, .048)	.041 (.038, .043)

### Grade and sex differences

To assess grade and sex differences in APSS scale scores, we calculated scores for the complete data set using factor score weights provided in AMOS (see Table 1). The factor scores are shown in Table 3, by grade and sex. These scores were then used as dependent variables in a two-way independent ANOVA with sex and grade entered as independent variables. For the *Mood Management* sub-scale there were no significant main effects, but there was a significant interaction, *F* (2, 1668) = 18.25, *p* < .001, *η*_*p*_^*2*^ = .021. Three Bonferroni corrected *t*-tests (.05 / 3 = .017) were carried out to compare boys and girls at each grade level. At Grade 5, boys (*M* = 1.95, *SD* = 0.90) reported significantly higher *Mood Management* scores than girls (*M* = 1.53, *SD* = 0.72), *t* (487) = 5.69, *p* < .001. There was no significant sex difference when comparing Grade 7 boys (*M* = 1.66, *SD* = 0.74) and girls (*M* = 1.74, *SD* = 0.90), nor when comparing Grade 9 boys (*M* = 1.64, *SD* = 0.74) and girls (*M* = 1.75, *SD* = 0.83).

**Table 3:** Means (Standard Deviations) for mood management and behavioural preoccupation, shown by sex and grade

Grade	Sex	Mood managament	Behavioural preoccupation
Grade 5	Boys	1.95 (0.90)^a^	1.82 (0.72)^d^
	Girls	1.53 (0.72)^a^	1.62 (0.70)^d^
	Overall	1.75 (0.84)	1.72 (0.72)
Grade 7	Boys	1.65 (0.74)^b^	1.80 (0.70)^e^
	Girls	1.74 (0.90)^b^	2.03 (0.85)^e^
	Overall	1.69 (0.82)	1.90 (0.78)
Grade 9	Boys	1.64 (0.74)^c^	1.94 (0.67)^f^
	Girls	1.75 (0.83)^c^	2.25 (0.79)^f^
	Overall	1.69 (0.78)	2.09 (0.75)

^a,d,e,f^Significantly different (*p* < .017)

^b,c^Not significantly different (*p* > .017)

For the *Behavioural Preoccupation* subscale, the same independent ANOVA analysis was repeated. This revealed a small, significant effect of sex, *F* (1, 1727) = 5.56, *p* = .001, *η*_*p*_^*2*^ = .006, and a small significant effect of grade level, *F* (2, 1727) = 19.77, *p* < .001, *η*_*p*_^*2*^ = .040. Both these effects were qualified by a significant interaction: *F* (2, 1727) = 10.48, *p* < .001, *η*_*p*_^*2*^ = .022. The same follow-up procedure was carried out as before, revealing that Grade 5 boys (*M* = 1.82, *SD* = 0.72) reported significantly higher *Behavioural Preoccupation* scores than girls (*M* = 1.62, *SD* = 0.70), *t* (520) = 3.26, *p* = .001. However, in Grade 7 the patterns were reversed, with boys (*M* = 1.80, *SD* = 0.70) reporting lower scores than girls (*M* = 2.03, *SD* = 0.85), *t* (624) = −3.82, *p* < .001. The same was true in Grade 9, boys (*M* = 1.94, *SD* = 0.67) reported lower scores than girls (*M* = 2.25, *SD* = 0.79), *t* (583) = −5.10, *p* < .001.

Using the full data set, analyses revealed that the two sub-scales were significantly correlated, *r* = .71, *n* = 1602, *p* < .001.

## Discussion

This study presents a new, short and easy-to-administer instrument with which to gauge adolescents' potential preoccupation with screen use across a broad range of screens and screen-based activities in non-clinical settings. In doing so it addresses the absence of such a measure and many of the concerns raised about current instrumentation [30].

The measure developed demonstrated good psychometric properties, with factorial validity shown through a number of accepted criteria for fit indexes: NFI = .922, CFI = .936, RMSEA = .059 (.056, .063) [43]. Internal reliability of both subscales was also strong (Cronbach alphas >.86). The strong factorial invariance of measurement across Grades 5 to 9 (i.e., 10 to 16 years), and across sex, are particular strengths since this means the measure can be used to examine developmental and sex differences during this adolescent period. Our data indicate that young adolescent males report higher levels of mood management using screens than girls, but by Grade 7 that difference disappears. With regards to behavioural preoccupation, the youngest adolescent males again reported higher levels than the youngest girls, but this effect was reversed by Grade 7 and older adolescent girls seemed to report an ever increasing degree of behavioural preoccupation. Differences in males’ and females’ screen use and screen use activities according to age have been documented in many studies (e.g., [2, 45, 46]) and our present results showing differences in levels and type of preoccupation with screens suggest that future research should be tailored to developmental stage as well as sex when seeking to unpack possible effects of screen engagement.

The APSS assesses both the mood management and behavioural preoccupation aspects of screen use that adolescents report. The APSS could prove useful as a way of understanding young people’s motivations and behaviours concerning their interactions with a wide range of screens. The APSS can also support the investigation of positive outcomes associated with screen use behaviors, particularly the use of screens to manage young people’s moods.

Unlike many other studies our data reflect screen use on academic **and** non-academic related activities before, during and after regular school hours. New mobile screen media are regularly accessed by young people throughout different times of the day and it is possible that their preoccupation with screens is a motivating factor for this. Conversely, such access may be a requirement of completing daily academic requirements in classrooms. Thus, asking questions which reflect usage throughout the waking day, including school time, is important.

The current study has a number of strengths. The sample was large and this allowed the factor structure of the APSS to be explored through exploratory factor analysis and then confirmed through confirmatory factor analysis. The sample was also generated from a large number of randomly sampled schools across a range of socioeconomic status areas. The instrument was also presented to adolescents in an online format so as to engage them. However, our results are based solely on self-report data. While the optimal recommended strategy is to use two or more sources [47], self-report is an effective means of obtaining an accurate insight into the subjective dispositions that can be difficult to obtain from third parties such as teachers and parents [48]. Moreover, because our data were collected to include the entire waking day, it was only the adolescents themselves who could supply this information.

There has been a need for a brief measure which takes into account all screens that adolescents use throughout their waking day, including mobile devices. Future studies should examine the adolescent’s levels of pre-occupation with screens in relation to specific screen activities and the amount of time they spend accessing screens. It is also important that further validation work is carried out using the APSS. Parent reports could be used to validate the behavioral preoccupation sub-scale, and one might expect the behavioural preoccupation sub-scale to be more strongly correlated with Fear of Missing Out (FoMO) [49] than the mood management sub-scale since FoMO involves the wish to be constantly connected to the internet in order to see what other people are doing. The behavioral subscale can also be expected to be more strongly correlated with gross screen time as objectively assessed using smartphone ‘on’ time [8].

## Conclusion

Young people can engage in many different ways with the entertainment, social media, and educational opportunities afforded by screen activity. Meta-analyses examining the effects for wellbeing of time spent on such activities suggest that there may only be small effects of screen use. However, more important than time spent on screen use per se may be a preoccupation with, and reliance on, screens. The present study presents the development of a new scale to assess such preoccupation with screens.
